# Emergence of Integrase Strand Transfer Inhibitor Resistance Following Treatment of Latent Tuberculosis Infection in a Patient With HIV

**DOI:** 10.1093/ofid/ofag448

**Published:** 2026-07-22

**Authors:** Emily Dyer, Shawnalyn W Sunagawa, Nichole Regan, Jennifer M Davis, Sara H Bares

**Affiliations:** Division of Infectious Diseases, University of Nebraska Medical Center, College of Medicine, Omaha, Nebraska, USA; Department of Pharmacy Practice and Science, University of Nebraska Medical Center, College of Pharmacy, Omaha, Nebraska, USA; Division of Infectious Diseases, University of Nebraska Medical Center, College of Medicine, Omaha, Nebraska, USA; Division of Infectious Diseases, University of Nebraska Medical Center, College of Medicine, Omaha, Nebraska, USA; Division of Infectious Diseases, University of Nebraska Medical Center, College of Medicine, Omaha, Nebraska, USA

**Keywords:** case report, HIV/TB coinfection, resistance

## Abstract

We present a case of emergent integrase strand transfer inhibitor resistance following rifamycin-based latent tuberculosis infection (LTBI) treatment in a patient with HIV-1This case highlights the risks associated with modifying antiretroviral therapy during LTBI treatment and illustrates how archived genotype results can directly impact clinical decision-making.

Although tuberculosis (TB) remains the leading cause of death globally among people with HIV (PWH), particularly in settings with a high burden of TB, the epidemiology differs in high-income countries where TB incidence is low and non-AIDS comorbidities predominate. Despite this lower incidence, screening for latent TB infection (LTBI) remains standard of care for all PWH in the United States regardless of epidemiologic risk, and treatment is recommended for those diagnosed with LTBI after exclusion of active disease due to the high risk of progression to active disease [1, 2].

Multiple trials have demonstrated that shorter rifamycin-based regimens have significantly higher completion rates (78–92%) compared with 9 months of isoniazid which is associated with completion rates of 50% or less in clinical practice [3–5]. As a result, rifamycin-based therapies are now the preferred regimens for LTBI treatment in PWH [1].

Although rifamycin-based therapy is recommended, rifamycins are potent inducers of cytochrome P450 enzymes (CYP; particularly CYP3A4 and CYP2C families), drug transporters (ie, P-glycoprotein [P-gp], breast cancer resistance protein [BCRP], and organic anion transporters [OAT]), and uridine diphosphate glucoronyltransferase 1A1 [UGT1A1]) [1]. This multipathway induction results in significant reductions in drug concentrations and impacts multiple classes of antiretroviral medications including nucleoside/tide reverse transcriptase inhibitors (NRTI) such as tenofovir alafenamide (TAF), non-nucleoside reverse transcriptase inhibitors, integrase strand transfer inhibitors (INSTIs), protease inhibitors, and the capsid inhibitor lenacapivir. As a result, antiretroviral treatment (ART) regimens frequently require dose adjustment or modification when rifamycin-based LTBI therapy is initiated.

Here, we present a case of emergent INSTI resistance following treatment of LTBI in a patient with recently diagnosed with HIV. Resistance was identified during thorough evaluation for eligibility to transition to long-acting injectable cabotegravir plus rilpivirine (LAI CAB/RPV).

## CASE REPORT

A 42-year-old male was diagnosed with HIV in August 2024 and initiated on bictegravir/emtricitabine/tenofovir alafenamide (BIC/FTC/TAF). Initial CD4 count was 897 cells/mm^3^ nd HIV-1 RNA was 14 000 copies/mL. Baseline genotype demonstrated wild-type virus without resistance-associated mutations (RAMs).

TB interferon gamma release assay was ordered as initial screening and resulted positive (TB1 Ag 9.78 IU/mL, TB2 Ag Response 9.78 IU/mL, reference range <0.35 IU/mL). Chest radiography was unremarkable, and the patient had no signs or symptoms of active TB infection, thus he was diagnosed with LTBI.

The patient was counseled regarding the importance of LTBI treatment. After discussion of all available treatment options and through shared clinical decision-making, he and his care team opted for a rifamycin-based treatment regimen. In mid-September 2024, he was prescribed a 3-month course of isoniazid 300 mg daily plus rifampin 600 mg daily plus pyridoxine 50 mg daily (3HR). Of note, rifampin was utilized in lieu of rifapentine as insurance did not approve rifapentine, so it was cost-prohibitive. Due to rifampin-associated interactions with both TAF (via P-gp induction) and BIC (via CYP3A, UGT1A1, and P-gp induction), his ART was adjusted to daily tenofovir disoproxil fumarate/emtricitabine plus twice-daily dolutegravir (TDF/FTC + BID DTG). HIV-1 RNA prior to LTBI treatment, after ∼6 weeks of BIC/FTC/TAF, had appropriately decreased to 33 copies/mL.

He filled a 1-month supply of both his LTBI and antiretrovirals in mid-September 2024 but was unable to fill his prescriptions in mid-October due to insurance issues, resulting in a 1-month interruption of both therapies; however, he restarted BIC/FTC/TAF in the interim. In mid-November, LTBI treatment was restarted with a new 3-month course; HIV-1 RNA at that time was <20 copies/mL.

He traveled to Guatemala in February 2025 and reported completing his LTBI treatment shortly after returning home. During this period, he reported running out of his ART for 2 days and taking 2 doses of a friend's medication, which he identified on pill chart as either elvitegravir/cobicistat/tenofovir alafenamide/emtricitabine or elvitegravir/cobicistat/tenofovir disoproxil fumarate/emtricitabine (EVG/COBI/TAF/FTC or EVG/COBI/TDF/FTC). He also acknowledged missing “some” evening doses of his twice-daily DTG during his LTBI treatment course.

**Figure 1.**
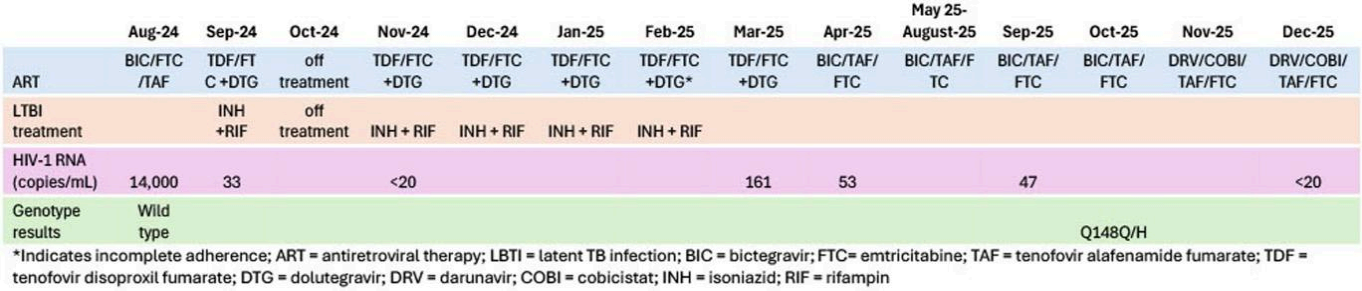
Timeline and clinical summary.

Following completion of LTBI therapy, he continued TDF/FTC+ BID DTG for 1 additional month to allow for more than sufficient time for rifampin washout and reversal of enzyme induction prior to transitioning back to once daily BIC/FTC/TAF in April 2025 [6]. HIV-1 RNA after completing LTBI treatment was 161 copies/mL. LTBI and ART regimens are outlined in Figure 1.

The patient later expressed interest in transitioning to LAI CAB/RPV and his case was reviewed for medical eligibility, consistent with our clinic's standard practice.

At the time of review, he was noted to have persistent low-level viremia (HIV-1 RNA ranging from 47 to 161 copies/mL), a recent history of rifamycin-based therapy for LTBI, and reported adherence gaps with twice-daily dosing of DTG. Given these findings, a proviral DNA archive genotype was obtained as part of the eligibility evaluation for LAI CAB/RPV prior to proceeding with any ART modification.

The proviral DNA archive genotype identified a Q148H RAM, which confers high-level resistance to raltegravir (RAL), EVG, and CAB, as well as low-to-intermediate reductions in susceptibility to DTG and BIC. The patient was informed that, due to the presence of the Q148H mutation, he would not be eligible for LAI CAB/RPV.

Given the Q148H integrase pathway mutation along with the persistent low-level viremia, he was transitioned to darunavir/cobicistat/tenofovir alafenamide/emtricitabine (DRV/COBI/TAF/FTC) and he achieved complete virologic suppression with an HIV-1 RNA of <20 copies/mL on this regimen. A regimen containing twice-daily DTG was not pursued due to prior adherence challenges with twice-daily dosing.

## DISCUSSION

Rifamycin-based treatments are preferred for LTBI in PWH, but certain rifamycins (most notably rifampin) are associated with potent CYP3A4 and drug transporter (eg, P-gp, BCRP, and OATP1B1/1BC) induction. As a result, patients on BIC/FTC/TAF are commonly transitioned to alternative ART regimens such as TDF/FTC + twice-daily DTG when rifampin is initiated [1]. Notably, not all rifamycin-based therapies necessitate this adjustment. Rifabutin, for example, is a moderate CYP3A4 inducer and does not require DTG dose modification [1, 7]. Rifapentine only requires DTG dose modification at certain dosing frequencies [8]. However, when rifampin is utilized, this dosage adjustment, while pharmacokinetically appropriate, introduces potential adherence challenges. Adherence to twice-daily medications has been shown to be significantly lower compared with once-daily medications [9]. Additionally, patients on single-tablet regimens (STRs) demonstrate greater adherence compared with multitablet regimens [10–12].

### Risks of Incomplete Adherence to Dolutegravir in the Setting of Rifamycin-based LTBI Therapy

Incomplete adherence to DTG, as observed in our patient, has been associated with the development of INSTI resistance [13]. The most common INSTI resistance mutations in persons with virologic failure on DTG-containing ART regimens are: R263K, G118R, N155H, and Q148H/R/K [14]. Q148H/R/K is a nonpolymorphic mutation that has been reported in patients prescribed RAL, EVG, CAB, and DTG [15]. Q148H/R/K alone confers minimal reduction in DTG susceptibility (median ∼0.8-fold reduction). However, this mutation typically occurs in combination with other mutations, particularly with G140A/S or E138K, and with at least 1 additional drug resistance mutation. These combinations confer a median 4.1-fold reduction in DTG susceptibility. This mutation also confers potential low-level resistance to BIC as well as high-level resistance to RAL, EVG, and CAB, limiting future therapeutic options.

We believe the Q148H mutation identified on proviral genotype developed due to incomplete adherence to BID DTG. Furthermore, since proviral DNA genotyping frequently fails to detect the full resistance profile in a given sample [16], along with the fact that Q148H rarely occurs in isolation and typically coevolves with compensatory mutations such as G140A/S or E138K, we are concerned that our patient may have acquired more than just the Q148H mutation.

### Alternative LTBI Regimens to Avoid Rifampin-Associated ART Modifications

For patients on BIC/FTC/TAF who cannot tolerate ART modifications required for rifampin-based LTBI treatment, alternative options exist. Isoniazid 5 mg/kg (max 300 mg) daily plus pyridoxine 25–50 mg daily for 6–9 months can be coadministered with any ART regimen and remains an alternative option [1]. This regimen is effective and reasonably well-tolerated but associated with suboptimal treatment completion rates due to its prolonged duration of therapy [3]. Another option is once-weekly isoniazid plus rifapentine, a less-potent CYP inducer than rifampin, for 12 weeks (3HP), which can be coadministered with once-daily DTG without dose adjustment. Although rifapentine results in less-potent CYP induction than rifampin, it should not be used with BIC-containing regimens due to significant reductions in BIC exposure [1].

The differential susceptibility of DTG versus BIC to rifamycin drug–drug interactions can be explained by their distinct metabolic pathways. DTG is primarily metabolized by UGT1A1 with only minor contributions from CYP3A, allowing its concentrations to be maintained with dose adjustment even in the setting of CYP3A induction by rifamycins [17]. In contrast, BIC is metabolized equally by both CYP3A and UGT1A1. Since rifamycins induce both of these pathways, the resulting dual induction produces a more profound and difficult-to-overcome reduction in overall BIC exposure, potentially precluding coadministration even with dose adjustment [18].

### Importance of a Standardized LAI Eligibility Screening Process

Because our patient expressed difficulty with daily medication adherence, LAI ART appeared to be a promising strategy. In our clinic, all patients being considered for LAI ART undergo standardized medical eligibility review. As part of this process, a proviral DNA genotype was obtained prior to regimen modification. While proviral genotyping has important clinical limitations [19] and is not obtained in all patients being evaluated for LAI CAB/RPV, it proved to be a useful tool in this case and this may not have been done had we not had a standardized process in place to screen for LAI eligibility.

### Importance of Low-Level Viremia as a Warning Sign

Low-level viremia should not be dismissed as clinically insignificant, particularly when considering a switch to 2-drug regimens such as LAI CAB/RPV. In 1 prospective study, low-level viremia (<200 copies/mL) on oral ART prior to transition to LAI CAB/RPV was associated with nonsustained viral suppression on LAI CAB/RPV, highlighting the need for careful evaluation before modifying ART [20]. Low-level viremia may be an indicator of underlying issues (eg, incomplete adherence or archived resistance) that could compromise outcomes on a 2-drug injectable regimen.

### Summary and Lessons Learned

As is commonly encountered, this patient with HIV/LTBI coinfection underwent multiple changes to ART, required twice-daily ART medication administration, and selected treatment with a rifamycin for the benefit of shorter LTBI treatment duration. The combination of these factors—regimen complexity, adherence challenges, and potential for reduced DTG exposure due to drug–drug interactions—created the conditions for emergence of resistance in this case. With increased interest from patients and providers in transitioning to LAI ART, the importance of LAI eligibility screening is becoming more certain. HIV care teams must pay close attention to ART changes, adherence reporting and potential for drug interactions that may decrease effectiveness of LAI ART and increase the chance of treatment failure. Additionally, cases of low-level viremia, such as in this case, must not be ignored as this can be the first signal of emerging resistance.
